# Transcriptional dynamics of transposable elements when converting fibroblast cells of *Macaca mulatta* to neuroepithelial stem cells

**DOI:** 10.1186/s12864-021-07717-9

**Published:** 2021-06-04

**Authors:** Dahai Liu, Li Liu, Kui Duan, Junqiang Guo, Shipeng Li, Zhigang Zhao, Xiaotuo Zhang, Nan Zhou, Yun Zheng

**Affiliations:** 1 Foshan Stomatology Hospital and School of Medicine, Foshan University, Foshan, Guangdong, 528000 China; 2 State Key Laboratory of Primate Biomedical Research; Institute of Primate Translational Medicine, Kunming University of Science and Technology, Kunming, Yunnan, 650500 China; 3 Faculty of Information Engineering and Automation, Kunming University of Science and Technology, Kunming, Yunnan, 650500 China; 4 State Key Laboratory of Genetic Engineering, Collaborative Innovation Center for Genetics and Development Institute of Plant Biology, School of Life Sciences, Fudan University, Shanghai, 200438 China

**Keywords:** Transposable element (TE), Endogenous retrovirus (ERV), Long terminal repeat (LTR), Transcription, Fibroblast, Induced pluripotent stem cell (iPSC), Neuroepithelial stem cell (NESC)

## Abstract

**Background:**

Transposable elements (TE) account for more than 50% of human genome. It has been reported that some types of TEs are dynamically regulated in the reprogramming of human cell lines. However, it is largely unknown whether some TEs in *Macaca mulatta* are also regulated during the reprogramming of cell lines of monkey.

**Results:**

Here, we systematically examined the transcriptional activities of TEs during the conversion of *Macaca mulatta* fibroblast cells to neuroepithelial stem cells (NESCs). Hundreds of TEs were dynamically regulated during the reprogramming of *Macaca mulatta* fibroblast cells. Furthermore, 48 Long Terminal Repeats (LTRs), as well as some integrase elements, of *Macaca* endogenous retrovirus 3 (MacERV3) were transiently activated during the early stages of the conversion process, some of which were further confirmed with PCR experiments. These LTRs were potentially bound by critical transcription factors for reprogramming, such as KLF4 and ETV5.

**Conclusion:**

These results suggest that the transcription of TEs are delicately regulated during the reprogramming of *Macaca mulatta* fibroblast cells. Although the family of ERVs activated during the reprogramming of fibroblast cells in *Macaca mulatta* is different from those in the reprogramming of human fibroblast cells, our results suggest that the activation of some ERVs is a conserved mechanism in primates for converting fibroblast cells to stem cells.

**Supplementary Information:**

The online version contains supplementary material available at (10.1186/s12864-021-07717-9).

## Introduction

Transposable elements (TEs) are abundant in mammalian genomes. According to the latest results of RepeatMasker (version open-4.0.5), the human and mouse genome consist of 52.6% and 45.0% TEs, respectively. TEs could largely be divided as two types, Class I retrotransposons and Class II DNA transposons [1]. While Class II DNA transposons use “cut and paste” mechanism to jump to different loci of the genome, Class I retrotransposons use “copy and paste” mechanism to spread through the genome which contributes to their abundance in the genome [1].

Endogenous retroviruses (ERVs) belong to Class I retrotransposons [1] and are remains of past retroviral infections [2]. ERVs normally consist of two flanking long terminal repeats (LTRs) on the two sides of central regions [1]. The numbers of LTRs are larger than those of ERVs since non-allelic homologous recombination could generate an additional copy of the so called “solo LTR” [1]. ERVs are normally bound by Krüppel associated box-Zinc Finger Proteins (KRAB-ZFPs) which then recruit TRIM28 for establishment of local heterochromatin [2–5]. ERVs play important roles in the development and differentiation processes of mammals [1, 2, 6]. Long interspersed nuclear elements (LINEs) and short interspersed nuclear elements (SINEs) have no flanking LTRs and also belong to Class I retrotransposons [1].

ERVs are actively regulated in stem cells and reprogramming of somatic cells. Pluripotent stem cells could be obtained from either embryonic stem cells [7] or induced pluripotent stem cells (iPSCs) through factor-mediated reprogramming [8]. It is widely reported that the transgenic expressions of four key transcription factors (TFs) (Myc, Klf4, Oct4 and Sox2) can convert somatic cells to generate induced pluripotent stem cells (iPSCs) [8–11]. These key TFs bind to the LTR of murine leukemia virus in mouse and LTR7 of HERV type-H (HERV-H) in human and activate the transcription of LTR-derived transcripts which is a critical mechanism in the generation of mouse and human iPSCs, respectively [3, 12–15]. Some LTR-derived transcripts are associated with enhancer regions and may contribute to the maintenance of pluripotency [14].

In comparisons to human and mouse, 49.33% of the genome of rhesus monkey *Macaca mulatta* are TEs. Although there have been many work in the TEs of human [12, 14, 16, 17] and mouse stem cells [14, 18], the study of TEs in monkey stem cells is very limited. Only a few studies of monkey stem cells were performed till now [19–21], but these studies did not pay attention to TEs. One recent study found that TE derived non-coding transcripts have conserved expression patterns in stem cells of four primates [22].

To enhance our understanding of TEs in the reprogramming of iPSCs in *Macaca mulatta*, we generated a set of time series RNA-Seq profiles when converting *Macaca mulatta* fibroblast cells to neuroepithelial stem cells (NESCs). Through bioinformatics analysis of the obtained RNA-Seq profile, we identified hundreds of TEs that are dynamically regulated in the induction process of NESCs from fibroblast cells. Furthermore, our results indicate that 48 LTRs of *Macaca* endogenous retrovirus 3 family (MacERV3) are transiently activated in the reprogramming of fibroblast cells, potentially through key TFs, such as KLF4 and ETV5. We also validated the transient activation of two LTRs of MacERV3 with PCR experiments. These results provide new insight into the potential roles of LTRs in the generation of NESCs of *Macaca mulatta*.

## Results and discussions

### Inducing the fibroblast cells to neuroepithelial stem cells

We used a combination of three stem cell TFs, i.e., SOX2, OCT4 and KLF4, to convert fibroblast cells of *Macaca mulatta* to NESCs [20] (see “Materials and methods” section). We collected fibroblast cells at the third generation before conversion (named as RF-P3), and cells on day 5 (as RF-iN-d5), day 11 (as RF-iN-d11) after initiation of the conversion protocol. These three cell lines represent the original fibroblast cells and those at early stages when fibroblast cells converting to neuroepithelial stem cells. The RF-iN-d11 cells were further passaged and cultured on 5 mg/ml laminin (Gibco) coated plates in iNESC-M culture media. We then collected epithelial cell colonies at early passages 6 (as CHIR-P6) and later passages 23 (as CHIR-P23). After that, two ideal single-cell formatted colonies (named as C8-P7 and A9-P7, respectively) were collected. On and after CHIR-P6, the procedure for producing NESCs was regarded as being finished. Thus, CHIR-P6 and later cells were regarded as NESCs [20]. The total RNAs of these 7 obtained cell lines (with two replicates for each cell line) were sequenced with Illumina HiSeq 2000 sequencer and we obtained 11 to 49 million 2×100 bp pair-end reads from these RNA-Seq profiles.

### The gene expression patterns in the reprogramming of fibroblast cells

**Fig. 1:**
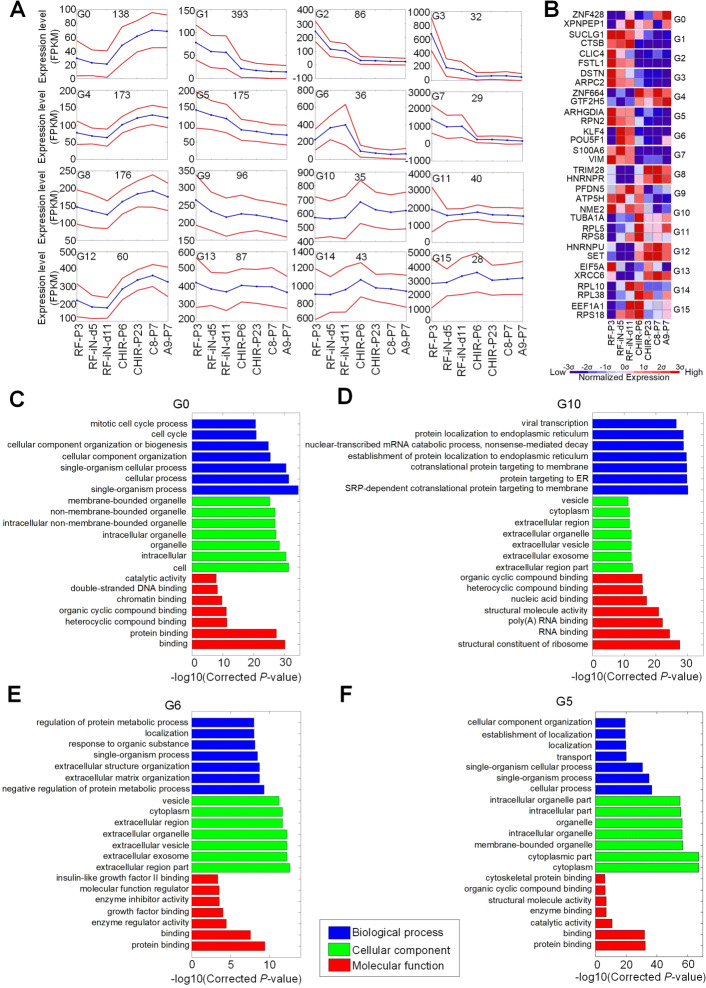
The clusters of gene with different expression patterns during the reprogramming of fibroblast cells in *Macaca mulatta.***(A)** The clusters of genes and their expression patterns at different stages of reprogramming. The numbers in the plots are the numbers of genes in the clusters. Blue lines indicate the average expression levels of genes in the cluster in different cell lines. Red lines indicate average expressions plus/minus standard deviations. **(B)** The expressions of two representative genes in each cluster of Part (A). **(C)** The most significantly enriched GO terms in the G0 cluster. **(D)** The most significantly enriched GO terms in the G10 cluster. **(E)** The most significantly enriched GO terms in the G6 cluster. **(F)** The most significantly enriched GO terms in the G5 cluster. In Part (C) to (F), the top 7 GO terms with the smallest multiple test corrected *P*-values in the three major GO categories were shown. The source data of Part (C) to (F) are available in Additional file 1: Table S4, S14, S10, and S9, respectively

We used the Cufflinks (v2.2.1) pipeline [23] to align the obtained RNA-Seq profiles to the genome of rhesus monkey and to quantify the abundance of genes at different stages of the reprogramming procedure. We obtained 40,705 genes after analyzing these 14 RNA-Seq profiles. These genes were filtered to keep 1627 genes with at least 20 FPKM in at least one of the 7 average values of two replicate and at least a standard deviation value of at least 20 FPKM in the 7 average values of two replicate (as listed in Additional file 1: Supplementary Table S1). These 1627 genes were clustered with the Self Organizing Map algorithm in the GeneCluster 2 package [24]. The obtained results were shown in Fig. 1a. The expressions of genes in Cluster G0 in Fig. 1a had a slight reduction at the third time point and then increased till the second last time point. On the contrary, the genes in Clusters G6 and G10 had increased expressions at the third and fourth time points, respectively. The expressions of genes in Cluster G5 gradually decreased in the whole reprogramming procedure.

We next analyzed the enriched GO terms of genes in the gene clusters and the significantly enriched (corrected *P*<0.05, Hypergeometric tests corrected with the Benjamini and Hochberg method [25]) GO terms of Clusters G0 to G15 were listed in Additional file 1: Table S2 to S17, respectively. The most significant GO terms of G0, G10, G6, and G5 are also shown in Fig. 1c to f, respectively.

The genes in G0 are upregulated after the fourth time point when the NESCs were established. As shown in Fig. 1c, two GO terms, i.e., mitotic cell cycle process and cell cycle, are enriched in G0, which is consistent with the unlimited proliferation property of stem cells. Several other enriched GO terms related to binding, such as protein binding, double stranded DNA-binding, intracellular organelle, organelle, cell, and chromatin binding, are also related to the proliferation.

The genes in G10 show a transient upregulation at the fourth time point, i.e., exactly when the NESCs were established. As shown in Fig. 1d, this cluster includes some GO terms related to endoplasmic reticulum (ER), RNA binding, poly(A) RNA binding, extracellular region, which might be related to the translations and transitions of proteins to extracellular regions. These results suggest that transitions of proteins to extracellular regions, potentially for communications between cells, are important when the NESCs were being established.

G6 is an important cluster and includes several key TFs, such as POU5F1 (also known as OCT4) and KLF4 (see Fig. 1b). This cluster has many enriched GO terms related to extracellular parts of cells (Fig. 1e), consistent with the physiological conversion of cell lines. Furthermore, binding and enzyme regulator activity, and regulation protein metabolic process are also enriched in G6, which is in accordance with the need to change the status of the cells. G6 also shows many GO terms related extracellular region, extracellular exosome, etc., suggesting that transitions of proteins to extracellular regions are active for fulfilling the reprogramming of fibroblast.

The genes in G5 show gradually decreased expression levels in the reprogramming of fibroblast cells. This cluster has some enriched GO terms related to cytoplasm, cytoplasmic part, cellular process, protein binding and binding (see Fig. 1f), suggesting that the genes in G5 are mainly involved in the normal growth of cells.

### The transcription dynamics of TEs in the reprogramming of fibroblast cells

**Fig. 2:**
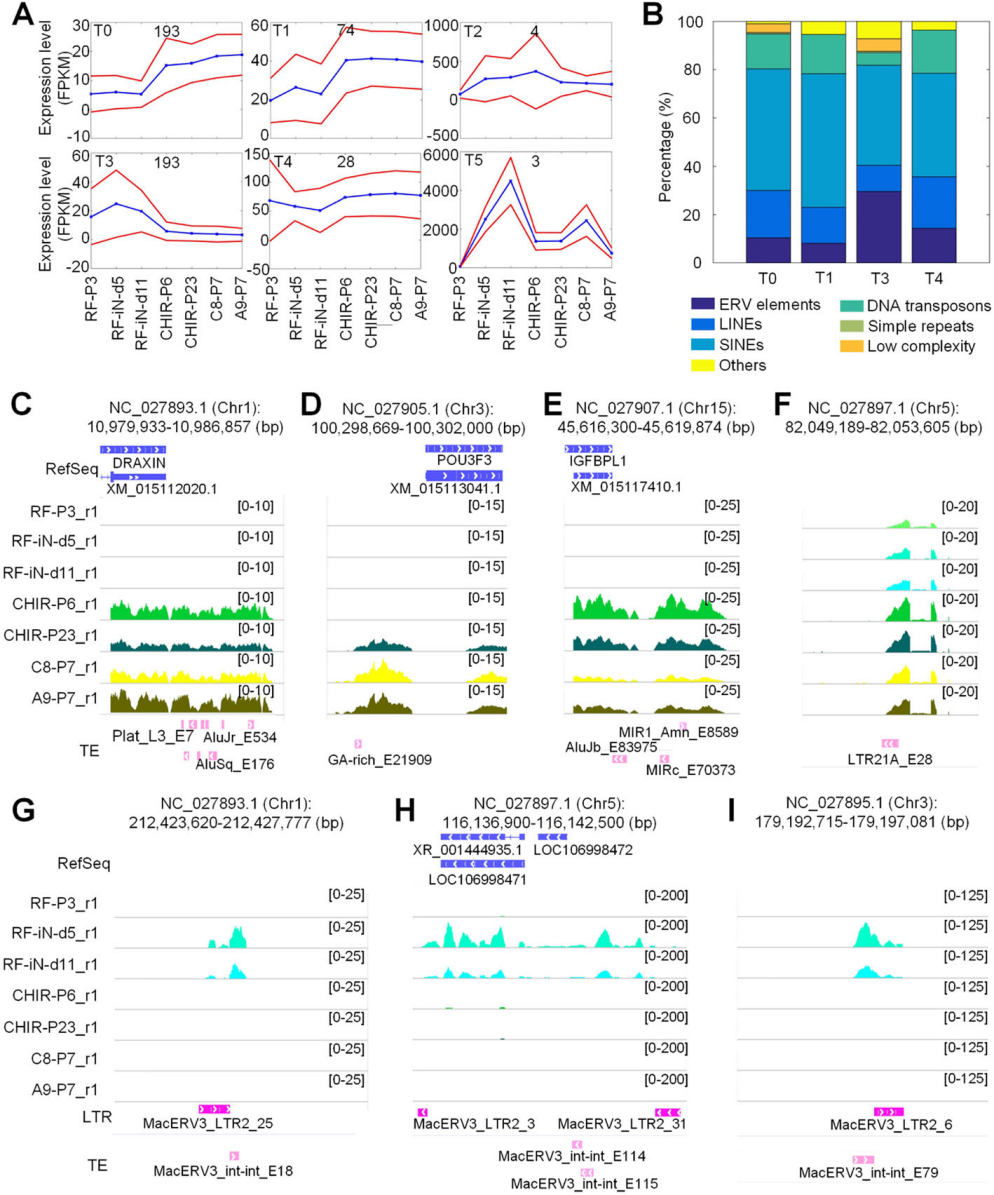
The clusters of TEs with different expression patterns during the reprogramming of fibroblast cells in *Macaca mulatta*. **(A)** The clusters of TEs and their expression patterns at different stages of reprogramming. **(B)** The categories of TEs in different clusters. Blue lines indicate the average expression levels of genes in the cluster in different cell lines. Red lines indicate average expressions plus/minus standard deviations. **(C) - (D)** Several TEs that belong to the Cluster T0 of Part (A). **(E) - (F)** Several TEs that belong to the Cluster T1 in Part (A). **(G) - (I)** Several MacERV3 integrase elements that belong to the Cluster T3 in Part (A). In Part (C) to (I), one of the two replicated RNA-Seq profiles for each lines were shown

Because TEs contribute to the reprogramming of cell lines, we carefully examined the transcriptional patterns of TEs in the reprogramming of fibroblast cells in *Macaca mulatta*. After removing TEs with very low expression levels and constant expression levels, we kept 2067 TEs with abundance of at least 5 FPKM in at least one of the RNA-Seq profiles and with standard deviations of at least 5 FPKM in the 14 profiles. Then, TEs that overlapped to coding genes were excluded and finally 495 TEs were identified as dynamically regulated TEs during the reprogramming of fibroblast cells (as listed in Additional file 1: Table S18). These 495 TEs were then grouped to 6 clusters based on their expression patterns in the reprogramming of fibroblast cells with the SOM algorithm in the GeneCluster 2 package [24] (see Fig. 2a).

These 6 clusters mainly have three expression patterns. The first group, i.e., T0 to T1, shows gradual increases of expression during the conversion process. The second group, i.e., T3, has a clear increase of expression at the second and third time point during the conversion process, but the expressions of these TEs were completely silenced from the fourth time point (CHIR-P6), i.e., after the fibroblast cells were converted to NESCs. The third group (T2, T4 and T5) only shows slight fluctuations or has limited TEs.

As shown in Fig. 2b, approximately 80% TEs in Clusters T0, T1, T3 and T4 are Class I retrotransposons, i.e., ERV elements, LINEs and SINEs. The percentage of LTR/ERV elements in the T3 cluster was significantly larger than other clusters (Fig. 2b) and thirty two of the 55 LTR/ERV elements in Cluster T3 are MacERV3 integrase elements (Additional file 1: Table S18).

As shown in Fig. 2c, a region near DRAXIN on Chr2 included 9 TEs, all of which showed gradually increased expression during the reprogramming procedure of fibroblast and were included in the Cluster T0 in Part A of Fig. 2. A low complexity TE (GA-rich_E21909) near POU3F3 was only expressed in the last three cell lines (Fig. 2d) and was included in Cluster T0 as well. Similarly, three TEs near IGFBPL1 were only expressed in the last 4 cell lines and were grouped in Cluster T1 (Fig. 2e). An LTR (LTR21A_E28) on Chr5 had limited expressed in fibroblast and was gradually upregulated during the reprogramming progressed (Fig. 2f) and was grouped in Cluster T1 too.

We examined several MacERV3 integrase elements in Cluster T3 as well. As shown in Fig. 2g, MacERV3_int-int_E18 was only expressed in the second and third cell lines, i.e., in the early stages of reprogramming of fibroblast. MacERV3_LTR2_25 beside MacERV3_int-int_E18 was not found in the 495 dynamically regulated TEs, but showed similar expression patterns as MacERV3_int-int_E18. Similarly, MacERV3_int-int_E114/E115 in Fig. 2h and MacERV3_int-int_E79 in Fig. 2i were detected in the second and third cell lines, however the LTRs beside these MacERV3 integrase elements were not identified as dynamically regulated TEs.

### LTRs of macERV3 are transiently activated in the reprogramming of fibroblast cells

**Fig. 3:**
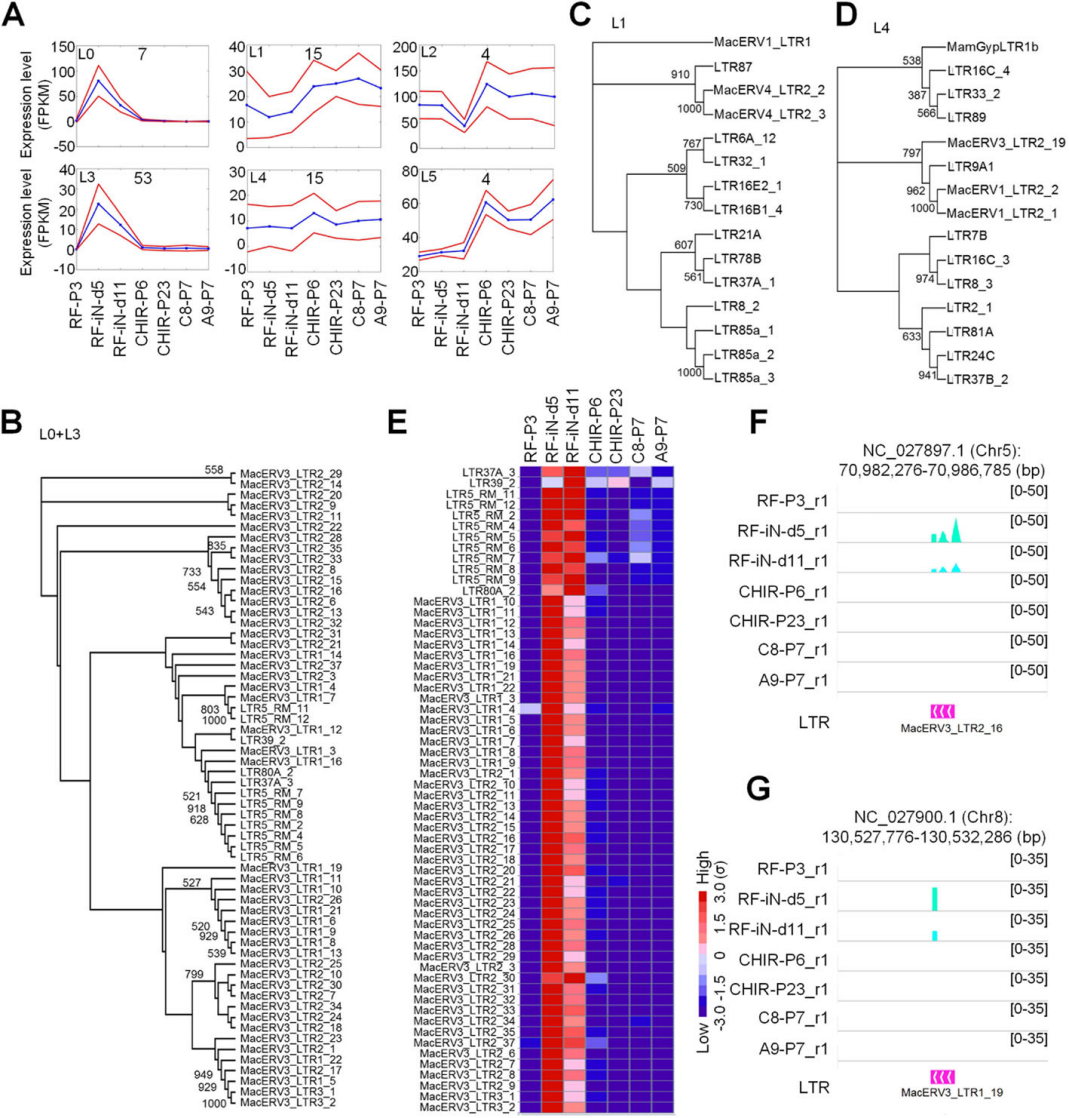
The clusters of LTRs with different expression patterns during the reprogramming of fibroblast cells in *Macaca mulatta*. In Part (B) to (D), the numbers in the trees are bootstrap values greater than 500 (50%). **(A)** The clusters of LTRs and their expression patterns at different stages of reprogramming. Blue lines indicate the average expression levels of genes in the cluster in different cell lines. Red lines indicate average expressions plus/minus standard deviations. **(B)** The phylogenetic tree of LTRs in the Clusters L0 and L3 of Part (A). **(C)** The phylogenetic tree of LTRs in the Cluster L1 in Part (A). **(D)** The phylogenetic tree of LTRs in the Cluster L4 in Part (A). **(E)** The expression levels of LTRs in the Clusters L0 and L3 of Part (A). **(F) - (G)** The expressions of two solo LTRs in the 7 cell lines. One of the two replicated RNA-Seq profiles for each lines were shown

More than 30 MacERV3 integrase elements were activated at the second and third time point as in Cluster T3 of Fig. 2a, but only one LTR of MacERV3 was identified in the same cluster of TEs (see Additional file 1: Table S18). We guessed that LTRs could not be sensitively detected with the featureCounts program because some LTRs were of relatively smaller sizes. Therefore, we recalculated the abundance of all LTRs with a more sensitive method to take all reads that covered the LTRs into account (see “Materials and methods” section), and kept LTRs with average expression levels of at least 5 FPKM in at least one of the 7 time points and standard deviation values of at least 5 FPKM. After removing LTRs overlapped to coding genes, we finally obtained 98 LTRs (as listed in Additional file 1: Table S19) that were clustered with the SOM algorithm in the GeneCluster 2 package [24]. As shown by the LTR clusters in Fig. 3a, we found that L0 and L3 with a total of 60 LTRs were activated at the second and third time points but were silenced after the third time point, which had similar expression patterns to the MacERV3 integrase elements in the Cluster T3 in Fig. 2. Most of these 60 (48/60) LTRs were LTR1 and LTR2 of MacERV3s. Actually, MacERV3_LTR2_25 in Fig. 2g, MacERV3_LTR2_3 and MacERV3_LTR2_31 in Fig. 2h were grouped in Cluster L3 of LTRs in Fig. 3a, and MacERV3_LTR2_6 in Fig. 2i was included in Cluster L0 of LTRs in Fig. 3a. In summary, these results suggest that the LTRs and integrates of MacERV3s were transiently activated in the early stages when converting monkey fibroblast cells to NESCs, but their expression levels were decreased to approximately 0 after the reprogramming procedure is finished.

We performed phylogenetic analysis for LTRs in Clusters L0+L3, L1 and L4. As shown in Fig. 3b to d, LTRs in L0+L3 were mainly LTRs of MacERV3, but L1 and L4 include very diverse types of LTRs.

The LTRs in Clusters L0 and L3 have very clear activation at the second and third cell lines (Fig. 3e). As shown in Fig. 3f and G, two solo LTRs were activated at the second and third cell lines. In comparison, the LTRs in Cluster L4 show almost constant expressions during the reprogramming procedure (Additional file 2: Figure S1).

We then examined the genomic contexts of the 60 LTRs in Clusters L0+L3. We found that 10 of these 60 LTRs locate beside annotated genes (Additional file 1: Table S19), with distances of smaller than 5 thousand basepairs, and were potentially transcribed as parts of long transcripts; and the other 50 are solo LTRs. The expression levels of 9 of these 10 LTRs near genes are positively correlated with those of their adjacent genes (Additional file 1: Table S19), suggesting their promoter functions to neighboring genes. Furthermore, five of these 10 LTRs locate in the upstream regions of annotated genes and potentially serve as promoters of these genes (Additional file 1: Table S19).

### Validating the expressions of MacERV3 LTRs with PCR experiments

**Fig. 4:**
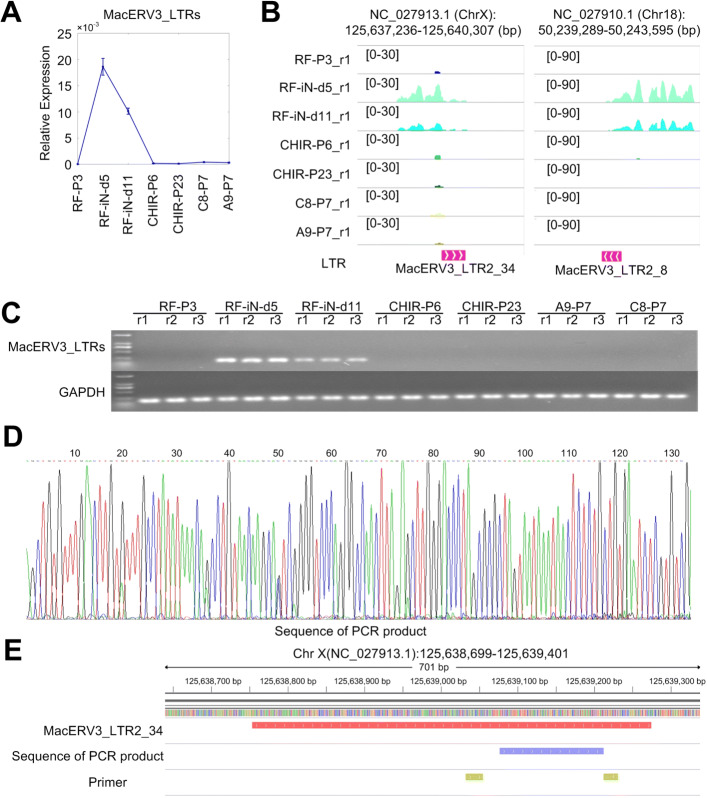
Validating the expressions of some MacERV3 LTRs with PCR experiments. **(A)** The relative expressions of 13 MacERV3 LTRs detected with the qRT-PCR experiments. **(B)** The expressions of the two MacERV3 LTRs in Part (A) detected with RNA-Seq profiles. One of the two replicated RNA-Seq profiles for each lines were shown. **(C)** The expressions of the LTRs in Part (A) examined with semi-quantitative RT-PCR in the samples used. GAPDH was used as an internal control. Three replicates (r1 to r3) were included for each of the 7 cell lines. (**D**) The sequence and scores of nucleotides of the PCR product. (**E**) The genomic loci of MacERV3_LTR2_34. The positions of the PCR product and primers were shown in different lanes below MacERV3_LTR2_34

To further confirm the transient expressions of MacERV3 LTRs at early stages of NESC conversion procedure, we selected 13 LTRs of MacERV3 (in Additional file 1: Table S20) for validation with PCR experiments with a set of primers shared by these LTRs (Additional file 2: Figure S2). These selected LTRs were successfully amplified in all of the samples of RF-iN-d5 and RF-iN-d11 cells. As shown in Fig. 4a, these LTRs showed clear activation at the early stages of the conversion procedure and reached maximal expressions in the cell line of RF-iN-d5, which was consistent to their expressions in the RNA-Seq profiles (as shown in Fig. 4b). The expression levels examined with gel image also have similar patterns as those detected by the qRT-PCR assays (Fig. 4c). We also sequenced the obtained product in the PCR experiments with Sanger sequencing. As shown in Fig. 4d and e, the obtained sequence located in the expected region between two primers from MacERV2_LTR2_34.

### KLF4 and ETV5 potentially activated MacERV3 LTRs in the reprogramming of fibroblast cells

**Fig. 5:**
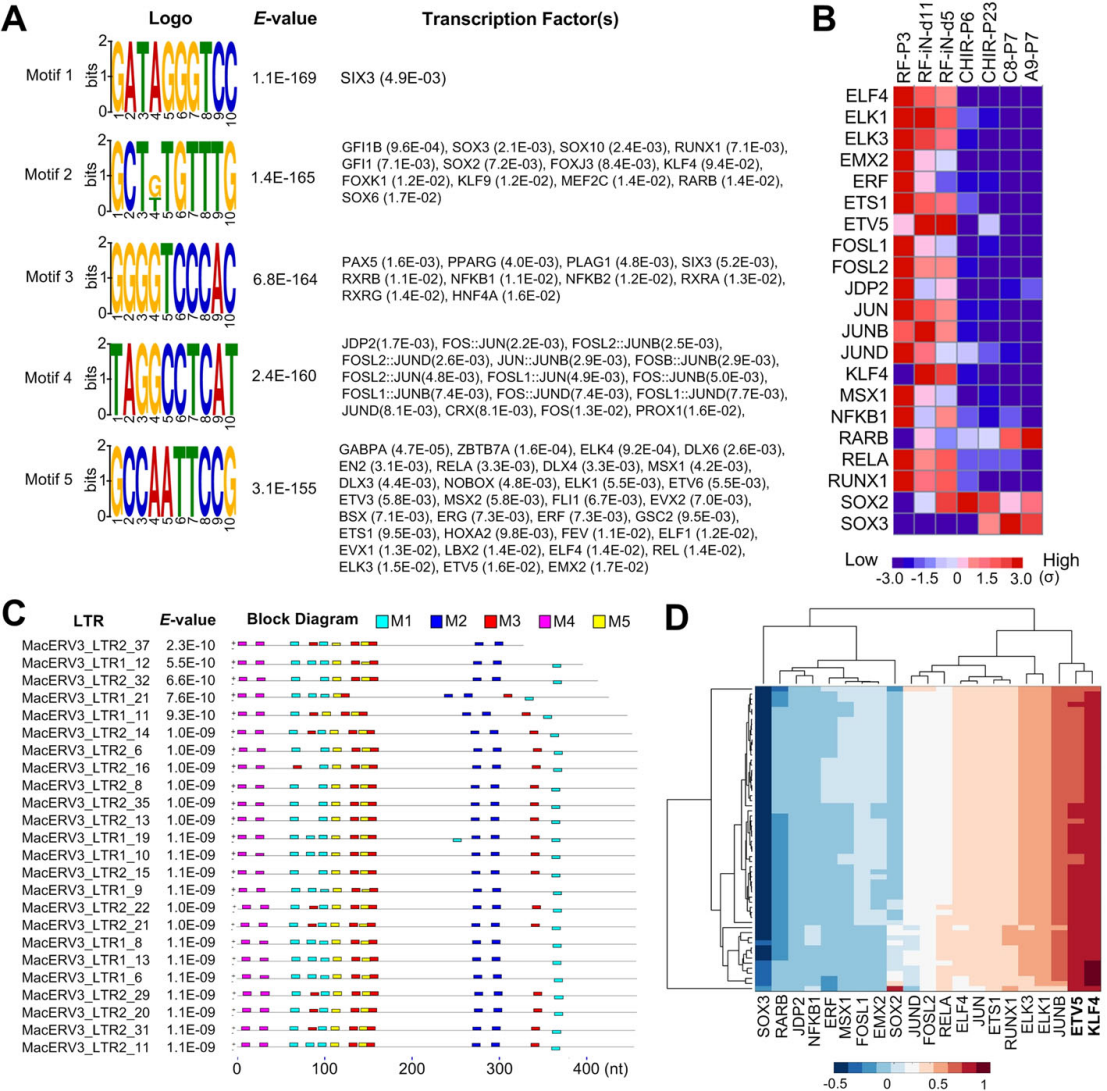
The enriched sequence motifs in the 60 LTRs activated at the early stages of reprogramming rhesus monkey fibroblast cells to NESCs. **(A)** The five motifs with the smallest *E*-values and their corresponding putative TFs. **(B)** The expression levels of the TFs in Part (A). Only those TFs with at least 5 FPKM in at least one of the 7 samples and standard deviation values of at least 5 FPKM were shown. **(C)** The distribution of motifs on some of the selected LTRs. M1 to M5 represents Motif 1 to Motif 5 in Part (A), respectively. **(D)** The bidirectional clustering of TEs and TFs in Part (B) based on the correlation coefficient values of their expression levels

Our results show that LTRs and integrase elements of MacERV3 are activated in the reprogramming of monkey fibroblast cells, and existing results in human and mouse show that LTRs contain *cis*-regulatory motifs of key TFs of stem cells [2–5, 12]. Therefore, we identified enriched *cis*-regulatory motifs in the activated LTRs and putative TFs that bind to these motifs with MEME [26]. As shown in Fig. 5a and c, we identified five significantly enriched motifs (*E*-values <10^−150^) from LTRs in Clusters L0 and L3 of Fig. 3a, and these motifs were related to some TFs with TOMTOM [27].

Since different members of the same TF family may share very similar *cis*-regulatory motifs, but only a few members may really be expressed and functional in the reprogramming of monkey fibroblast cells. We first filtered all putative TFs to keep 21 TFs with at least 5 FPKM in at least one of the 7 time points and standard deviation values of at least 5 FPKM (as shown in Fig. 5b). To further validate the putative regulatory relations between the remaining 21 TFs and the 60 MacERV3 LTRs in Cluster L0 and L3 of Fig. 3a, we calculated the correlation coefficients between the TFs and these MacERV3 LTRs, then performed a bidirectional hierarchical clustering using the obtained correlation coefficient matrix. As shown in Fig. 5d, KLF4 and ETV5 (ETS variant 5) had very high positive correlation coefficient values with almost all the MacERV3 LTRs examined, suggesting that KLF4 and ETV5 activated these MacERV3 LTRs in the early stages of the reprogramming of monkey fibroblast cells. Actually, the correlation coefficient values between the expression levels of KLF4 and the 48 LTRs of MacERV3 in Clusters L0 and L3 of Fig. 3a range from 0.875 to 0.998, all of which are very significant (*P*<0.01). The expression levels of ETV5 and the same 48 LTRs are also significantly correlated with correlation coefficient values from 0.749 to 0.923, and 47/48 of these values are significant (*P*<0.05).

KLF4 is one of the key TFs for inducing iPSCs [8–11]. Thus, it is reasonable that KLF4 activated the expressions of MacERV3 LTRs. Beside KLF4, ETV5 is also identified as a putative regulator of MacERV3 LTRs. ELK1 and ELK3 also show large positive correlation coefficient values with most MacERV3 LTRs examined. ETV5, ELK1 and ELK3 belong to the ETS TF family. The members of this family have been implicated in the development of different tissues as well as cancer progression. ETS genes also play important roles in the specification and differentiation of dopaminergic neurons in both *C. elegans* and olfactory bulbs of mice [28]. Our results suggest that the ETS members, ETV5, ELK1 and ELK3, might contribute to the reprogramming of monkey fibroblast cells by joining the activation of LTRs of MacERV3s.

**Fig. 6:**
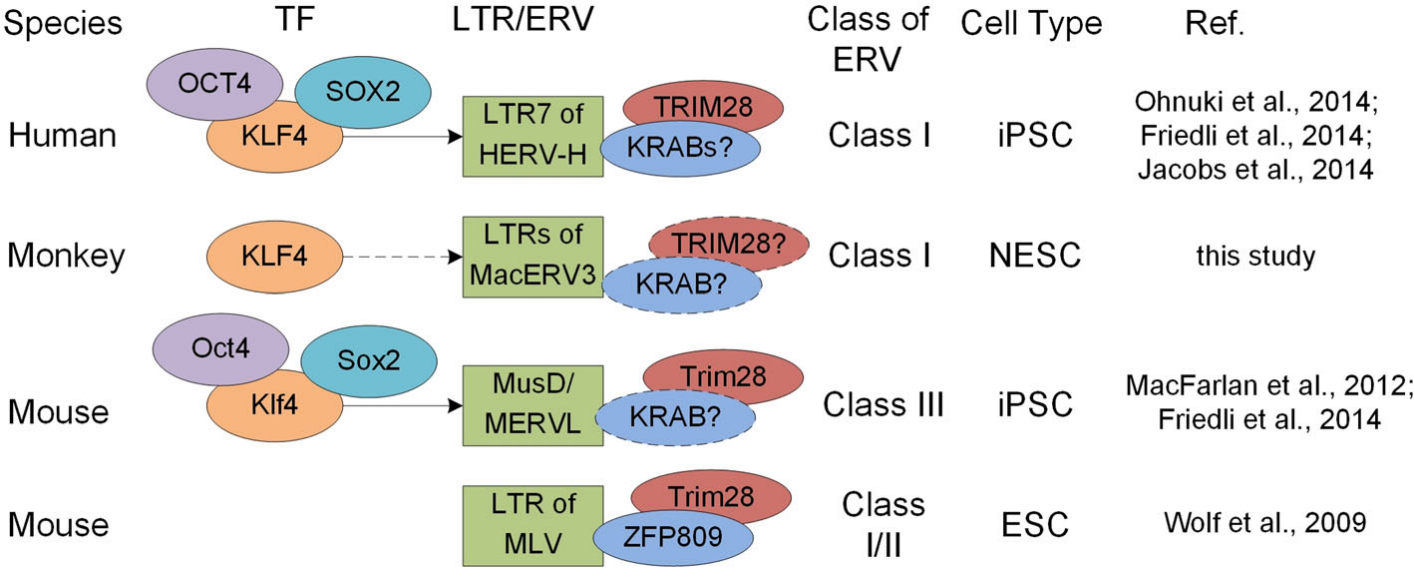
A brief summary of KLF4, LTR and KRAB-ZFP/TRIM28 regulatory systems in iPSCs, NESCs and ESCs of different vertebrates. Arrows from TFs to LTRs mean direct regulatory relations. Dashed arrow from KLF4 to LTRs of MacERV3 means predicted regulatory relations based on motif analysis with MEME and correlation coefficient between expression levels of KLF4 and these LTRs. “?”: The KRAB-ZFP concordant with TRIM28 in human and mouse iPSC generation and the recruitment of TRIM28 to LTRs of MacERV3 by an unknown KRAB for monkey are still to be verified

As shown in Fig. 6, when inducing human iPSCs, OCT3/4, SOX2, and KLF4 transiently hyperactivated LTR7s of Human ERV-H (HERV-H) through direct occupation on LTR7 sites [12, 13]. When producing mouse iPSCs, MuSD (Class II ERV) and MERVL (Class III ERV) were transiently activated in reprogramming to iPSCs [13]. Our results suggest that KLF4 activates LTRs and integrates of MacERV3 through its binding sites on LTRs.

As shown in Fig. 6, after ERVs were activated, KRAB-ZFPs bind to LTRs and recruit TRIM28 to induce hetereochromatin to silence the LTRs [3, 29]. For example, an murine KRAB-ZFP, ZFP809, represses murine leukemia virus (MLV) in embroynic stem cells and recruits Trim28 (also known as KAP1) to the LTRs of MLV [3]. In human, ZNF91 and ZNF93 were found to repress two retrotransposons SVA and L1, respectively [30]. The expression level of TRIM28 gradually increases in the reprogramming procedure of human CD34+ cells, putatively as a mechanism to repress HERVH that is transiently activated when the reprogrammed cells approach iPSC stage [2, 13]. We noticed that TRIM28 also showed gradually increased expression levels on and after the third cell line (RF-iN-d11) in the 7 cell lines of this study (see Fig. 1b and Additional file 1: Table S1), potentially to repress expressions of MacERV3 elements. In the future, it is thus interesting to further explore whether a KRAB-ZFP also binds to MacERV3 LTRs and recruits TRIM28 to these loci in the reprogramming of monkey fibroblast cells.

MacERV3, as well as HERV-H and MLV, belongs to the Class I ERV [2, 31]. In summary, our results suggest that KLF4 is the key transcription factor in activating Class I ERVs in *Macaca mulatta* during the reprogramming procedures of somatic cells toward iPSCs or NESCs. Subsequently, TRIM28 is potentially recruited by an unknown KRAB-ZFP to silence the MacERV3 elements.

## Conclusion

Our results show that hundreds of TEs have dynamic expression patterns during the reprogramming of fibroblast cells of *Macaca mulatta*. Forty eight LTRs of the MacERV3 family are activated at the early stages of the reprogramming procedure and depleted after the reprogramming is finished. The LTRs of MacERV3s share very similar sequence motifs that are potentially bound by several TFs such as KLF4 and ETV5. The expression levels of MacERV3 LTRs and KLF4/ETV5 are significantly correlated, suggesting that these TFs activate the expressions of LTRs of MacERV3 during the reprogramming of fibroblast cells of *Macaca mulatta*, and these MacERV3 elements were putatively silenced by TRIM28 after the reprogramming is finished.

## Materials and methods

### Cell lines and reprogramming procedures

Ear skin fibroblasts (named as RF-P3) of *Macaca mulatta* were obtained in our previous study [20]. RF-P3 was maintained in high glucose DMEM, 10% FBS and incubated at 37^∘^C, 5% CO_2_. The plasmids pMXs-SOX2, pMXsOCT4 and pMXs-KLF4 with rhesus monkey sequences were gifted from Hongkui Deng Lab at Peking University. The three concentrated retroviruses including OCT4/SOX2/KLF4 were mixed to twice infect monkey fibroblasts which were passaged 24 h before at 3×10^4^ cells per 35 mm dish. At the 3rd day after infection (piD3), fibroblasts were harvested by trypsin digestion and replated on laminin-coated plates at 1×10^5^ cells in one well of a 6-well-plate pre-coated with 5 mg/ml laminin (Gibco) in 3 ml induction medium (iNSC-M) supplemented with 5 *μ*M Y27632 (StemRD) and 1 *μ*M valproic acid (VPA) (TOCRIS). iNSC-M is composed of Neurobasal media with 1xB27 (Gibco), 1xN2 (Gibco), 1XNEAA (Gibco), 1% Glutmax (Gibco), 0.1 *μ*M 2-mercaptoethanol (Sigma), 50 mg/ml Vitamin C (Sigma), 3 *μ*M CHIR99021 (Cellagen technology), 5 *μ*M SB431542 (Cellagen technology) and 10 ng/ml bFGF (Millipore, GF003AF). Y27632 and VPA were removed from the media on the 5th and 7th day, respectively. Cells collected on the 5th day were named as RF-iN-d5. On the 7th day, the confluent fibroblasts were digested into single cells for further passaging at the ratio of 1:3 and cultured in iNSC-M without Y27632 and VPA until the 11th day. Cells collected on the 11th day were named as RF-iN-d11.

Induced epithelial cell colonies at the 11th day were gently detached from the plates with pipette pressure for further passaging and cultured on 5 mg/ml laminin (Gibco) coated plates in iNESC-M culture media. Then, we collected epithelial cell colonies at early passages 6 (named as CHIR-P6) and later passages 23 (named CHIR-P23). The iNESC-M is composed of Neurobasal media with 1xB27, 1xN2, 1XNEAA, 1% Glutmax, SB431542, 50 mg/ml Vitamin C, 10 ng/ml bFGF and 1000 U/ml hLIF (Millipore).

The induced epithelial cell colonies at passages 23 were dissociated into single cells with trypsin, and cultured on 5 mg/ml laminin (Gibco) coated 96-well-plates in iNESC-M culture media. Ultimately, we have obtained two ideal single-cell formatted colonies, named as C8-P7 and A9-P7. 0.025% trypsin (Sigma) was used to digest iNESCs for encouraging cell propagation when passaging. The cells were routinely passaged to 1:8 to 1:16 ratios every 3-4 days in the iNESC-M. Two biological replicates were collected for each of the seven cell lines.

### Extraction of total RNAs and RNA-Seq

The total RNAs of the 7 selected cell lines, i.e., RF-P3, RF-iN-d5, RF-iN-d11, CHIR-P6, CHIR-P23, C8-P7 and A9-P7, were extracted with the Trizol reagent (product No. 15596026) (Thermo Fisher, MA, USA) according to the manufacturer’s protocol. Based on the ratio of the optical density at 260 nm to that at 280 nm (OD260/280), the integrities of RNAs were checked by using an ultraviolet spectrophotometer (Hoefer, MA, USA). And then in view of visual comparison of the 18S and 28S ribosomal RNAs, the integrities of RNAs were also assessed by electrophoresis in a denaturing formaldehyde agarose gel. The total quantities of RNA samples with OD260/280 between 1.8 and 2.0 were examined. Samples with at least 20 *μ*g were selected for preparation of RNA-Seq libraries. 20 *μ*g total RNAs dissolved in 35 *μ*l were used to prepare ribo-depleted RNA-Seq libraries according to the manufacturer’s protocol. The RNA-Seq libraries were then sequenced by using the Illumina HiSeq 2000 sequencer with a 2×100 bp pair end mode. The 14 obtained RNA-Seq profiles were deposited into NCBI GEO database under the series accession number GSE137692.

### Calculating the abundance of genes and TEs

The 14 RNA-Seq profiles were aligned with HISAT2 (v2.1.0) [32] using the options of “-p 16 –dta-cufflinks -q –un-conc” to the genome of *Macaca mulatta* downloaded from the NCBI Genome Database (v5.0). The annotation of the RefSeq genes were used in the assembly of transcripts and genes with Cufflinks 2 (v2.2.1) [23]. Then, the abundance of genes was estimated with the Cufflinks pipeline (v2.2.1) using the default parameters [23]. The expression levels of genes for these samples are highly reproducible (Additional file 2: Figure S3), therefore the latter analyses use the average values of two replicate samples. Finally, 1627 genes were kept in further analysis by keeping those with average expression levels of at least 20 FPKM (Fragments Per Kilo basepairs per Million sequencing reads) in at least one of the 7 time points and at least standard deviation values of at least 20 FPKM (as listed in Additional file 1: Supplementary Table S1). These 1627 genes were clustered with the Self Organizing Map (SOM) algorithm implemented in the GeneCluster 2 package [24].

To calculate the abundance of TEs, we used the featureCounts program [33] using the options of “-p -T 4 -f -O -M –fraction –minOverlap 30” to calculate the count values of TEs reported by RepeatMasker (version open-4.0) [34]. Then, we used a self-developed program to calculate the FPKM values of TEs with the count values generated by featureCounts. The TEs with more than 10 FPKM in at least one of the 7 time points and standard deviation of more than 5 FPKM were kept and compared to the coding genes. TEs that overlapped with coding genes were excluded from further analysis. Finally, 495 remaining TEs (in Additional file 1: Table S18) were used to perform a clustering analysis using the SOM algorithm implemented in the GeneCluster 2 package [24].

To calculate the abundance of LTRs, we used the “bedtools genomecov” command of bedtools [35] to calculate the genome coverage of the RNA-Seq libraries. Then, a self-developed program was used to calculate the FPKMs (Fragments Per Kilo basepairs and per Million sequencing tags) of LTRs in the genome of *Macaca mulatta*, using the genome coverage results of RNA-Seq libraries. Briefly, we obtained the sum of the number of reads covering each position of LTRs. Then, this total overage number times 10^9^ was divided by the length of reads (in nt) times the length of LTRs (in nt) times total reads number in the library to obtain the FPKM values of LTRs. Next, the LTRs with more than 5 FPKM in at least one of the 7 time points and standard deviation of more than 5 FPKM were kept and compared to the coding genes. LTRs that overlapped with coding genes were excluded from further analysis. Finally, 98 remaining LTRs (in Additional file 1: Table S19) were used to perform a clustering analysis using the SOM algorithm implemented in the GeneCluster 2 package [24].

### GO term analysis for gene clusters with different expression patterns

The genes in different clusters were input into the KOBAS2 web server [36], respectively. The enrichments of GO terms were evaluated with the hypergeometric tests. The GO terms with multiple test corrected *P*-values (using the Benjamini and Hochberg method [25]) smaller than 0.05 were regarded as significant GO terms. The significant GO terms of gene clusters G0 to G15 were listed in the Additional file 1: Tables S2 to S17, respectively.

### Correlation analysis of transcriptional factors and LTRs

The expression levels of transcriptional factors that have significant binding motifs in the selected LTRs and the expression levels of selected LTRs were used to calculate a correlation coefficient matrix. A hierarchical bidirectional clustering was performed using the obtained correlation coefficient matrix.

### Validation of some LTRs with PCR experiments

As shown in Additional file 1: Table S20 and Additional file 2: Figure S2, 13 MacERV3 LTRs that were activated in the cell line of RF-iN-d5 (from the C0 or C3 cluster in Fig. 3) were selected for validation. We prepared three replicates of cells for each of the 7 cell lines, i.e., RF-P3, RF-iN-d5, RF-iN-d11, CHIR-P6, CHIR-P23, C8-P7 and A9-P7. The total RNAs of three replicates of the seven cell lines were retrieved with the TRIzol Reagent (product No. 15596026) (Thermo Fisher, MA, USA). Then, the high quality total RNAs were used to obtain cDNA library with PrimeScript RT reagent Kit (product No. RR047A) (Takara, Shiga, Japan). Next, we performed quantitative real-time PCR (qRT-PCR) to detect the expressions of the four selected LTR elements using TB Green Premix Ex Taq II kit (product No. RR820A) (Takara, Shiga, Japan) with CFX96 Real-Time PCR Detection System (BioRAD, CA, US). The primers used in the experiments were listed in Additional file 1: Table S21. Three qRT-PCR experiments were performed for each replicate of the 7 cell lines and the average values of these three experiments were used as the value of this replicate. In all experiments, GAPDH was used as control to calculate the relative expressions of selected LTRs.

Meanwhile, in order to view expressions of selected LTR elements in gel, we performed PCR in three different replicates for each of the seven cell lines. We applied 2 ×TSINGKE Master Mix (product No. TSE004) (TSINGKE, Beijing, China) to amplify the selected LTR elements products using the following conditions, 95^∘^C for 5 min, 95^∘^C for 30 s, 60^∘^C for 30 s, 72^∘^C for 20 s, 25 cycles, and 72^∘^C for 5 min.

## Supplementary Information

**Additional file 1** Supplementary Table S1. The expression levels of 1627 dynamically expressed genes in the reprogramming procedure of rhesus monkey fibroblast cells toward neuroephithelia stem cells.
Supplementary Table S2 to S17. The enriched GO terms of genes in Cluster G0 to G15 of Figure 1A, respectively.
Supplementary Table S18. The expression levels of 495 dynamically expressed TEs in the reprogramming procedure of rhesus monkey fibroblast cells toward neuroephithelia stem cells.
Supplementary Table S19. The expression levels of 98 dynamically expressed LTRs in the reprogramming procedure of rhesus monkey fibroblast cells toward neuroephithelia stem cells.
Supplementary Table S20. The 13 MacERV3 LTRs that were selected for PCR verification.
Supplementary Table S21. The primers used in this study.

**Additional file 2** Supplementary Figure S1. The expression levels of LTRs in the Cluster L4 of Figure 3A.
Supplementary Figure S2. The primers used to validate the expressions of 13 MacERV3 LTRs. (A) The alignment of the forward primer MacERV3_LTR2_34_F and the 13 MacERV3 LTRs. (B) The alignment of the reverse primer MacERV3_LTR2_34_R and the 13 MacERV3 LTRs. (C) The precise loci of the two primers in one of the selected MacERV3 LTRs, MacERV3_LTR2_34.
Supplementary Figure S3. The scatter plots of log2 scaled abundance (FPKM + 1) of genes. The correlation values (R) and *P*-values (*P*) were calculated by using the corrcoef function in MatLab. (A) The scatter plot for the two biological replicates of the RF-P3 cell line. (B) The scatter plot for the two biological replicates of the RF-iN-d5 cell line. (C) The scatter plot for the two biological replicates of the RF-iN-d11 cell line. (D) The scatter plot for the two biological replicates of the CHIR-P6 cell line. (E) The scatter plot for the two biological replicates of the CHIR-P23 cell line. (F) The scatter plot for the two biological replicates of the C8-P7 cell line. (G) The scatter plot for the two biological replicates of the A9-P7 cell line.

## Data Availability

The 14 RNA-Seq profiles generated in this study were available in the NCBI GEO database with the series accession number GSE137692 and in the NCBI SRA database with the series accession number SRP222479. Declarations

## Abbreviations

DRAXIN: Dorsal inhibitory axon guidance protein
ELK1: ETS transcription factor ELK1
ELK3: ETS transcription factor ELK3
ERV: Endogenous retrovirus
ETV5: ETS variant transcription factor 5
FPKM: Fragments per kilo basepairs per million sequencing reads
GO: Gene ontology
IGFBPL1: Insulin like growth factor binding protein like 1
iPSC: Induced pluripotent stem cells
KLF4: Kruppel like factor 4
KRAB-ZFP: Krüppel associated box-zinc finger protein
LINE: Long interspersed nuclear element
LTR: Long terminal repeat
Myc: Myelocytomatosis oncogene
NESC: Neuroepithelial stem cell
Oct4: Also known as POU5F1
POU3F3: POU class 3 homeobox 3
POU5F1: POU class 5 homeobox 1
SINE: Short interspersed nuclear element
SOM: Self-organizing map
Sox2: SRY-box transcription factor 2
TE: Transposable element
TF: Transcription factor.
