# Supplementary material for: Transcriptional dynamics of transposable elements when converting fibroblast cells of Macaca mulatta to neuroepithelial stem cells
Source: BMC Genomics. 2021 Jun 4;22(Suppl 3):405. doi: 10.1186/s12864-021-07717-9 (PMC8176597; doi:10.1186/s12864-021-07717-9)
Supplement: Supplementary file 2 — Additional file 2 Supplementary Figure S1. The expression levels of LTRs in the Cluster L4 of Figure 3A. Supplementary Figure S2. The primers used to validate the expressions of 13 MacERV3 LTRs. (A) The alignment of the forward primer MacERV3_LTR2_34_F and the 13 MacERV3 LTRs. (B) The alignment of the reverse primer MacERV3_LTR2_34_R and the 13 MacERV3 LTRs. (C) The precise loci of the two primers in one of the selected MacERV3 LTRs, MacERV3_LTR2_34. Supplementary Figure S3. The scatter plots of log2 scaled abundance (FPKM + 1) of genes. The correlation values (R) and P-values (P) were calculated by using the corrcoef function in MatLab. (A) The scatter plot for the two biological replicates of the RF-P3 cell line. (B) The scatter plot for the two biological replicates of the RF-iN-d5 cell line. (C) The scatter plot for the two biological replicates of the RF-iN-d11 cell line. (D) The scatter plot for the two biological replicates of the CHIR-P6 cell line. (E) The scatter plot for the two biological replicates of the CHIR-P23 cell line. (F) The scatter plot for the two biological replicates of the C8-P7 cell line. (G) The scatter plot for the two biological replicates of the A9-P7 cell line. [file 12864_2021_7717_MOESM2_ESM.pdf]

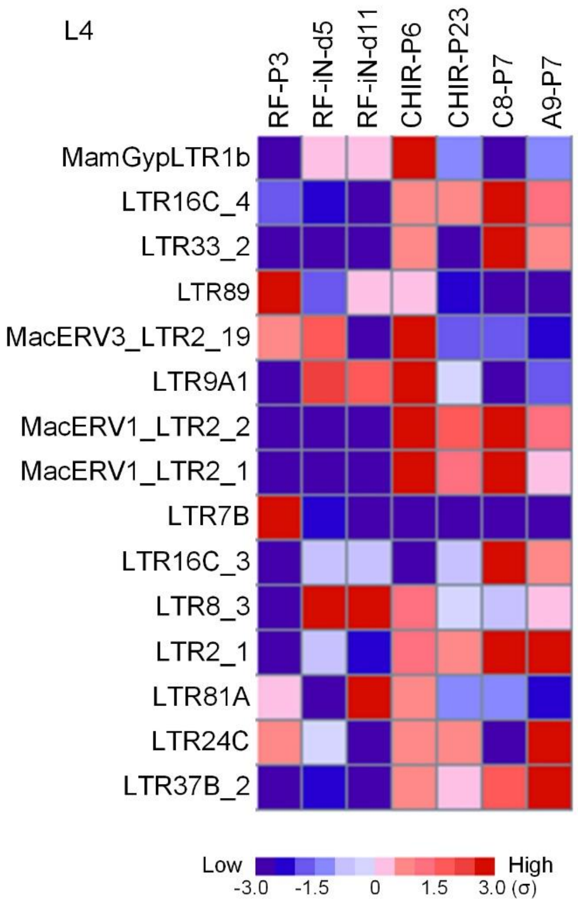

Figure S1. The expression levels of LTRs in the Cluster L4 of Figure 3A.

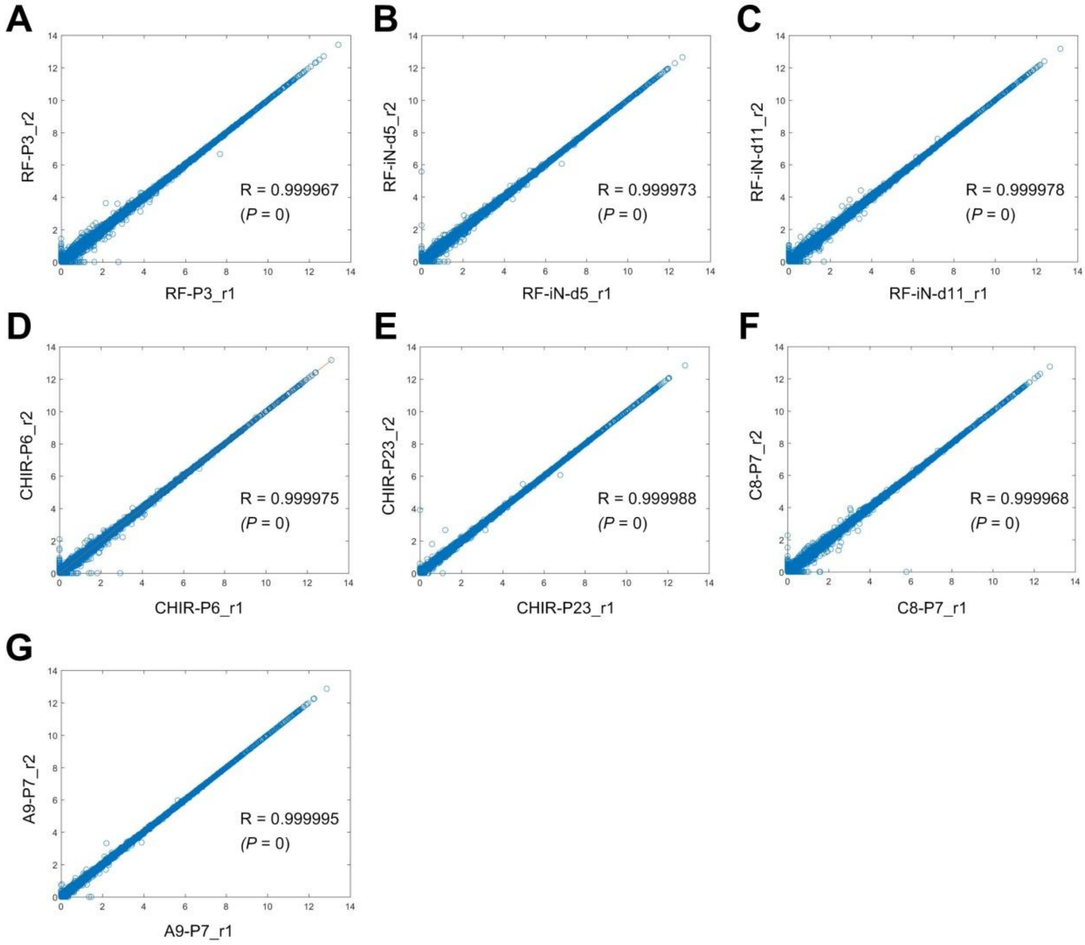

Figure S2. The scatter plots of log2 scaled abundances (FPKM + 1) of genes. The correlation values (R) and *P*-values (*P*) were calculated by using the corrcoef function in MatLab. (A) The scatter plot for the two biological replicates of the RF-P3 cell line. (B) The scatter plot for the two biological replicates of the RF-iN-d5 cell line. (C) The scatter plot for the two biological replicates of the RF-iN-d11 cell line. (D) The scatter plot for the two biological replicates of the CHIR-P6 cell line. (E) The scatter plot for the two biological replicates of the CHIR-P23 cell line. (F) The scatter plot for the two biological replicates of the C8-P7 cell line. (G) The scatter plot for the two biological replicates of the A9-P7 cell line.

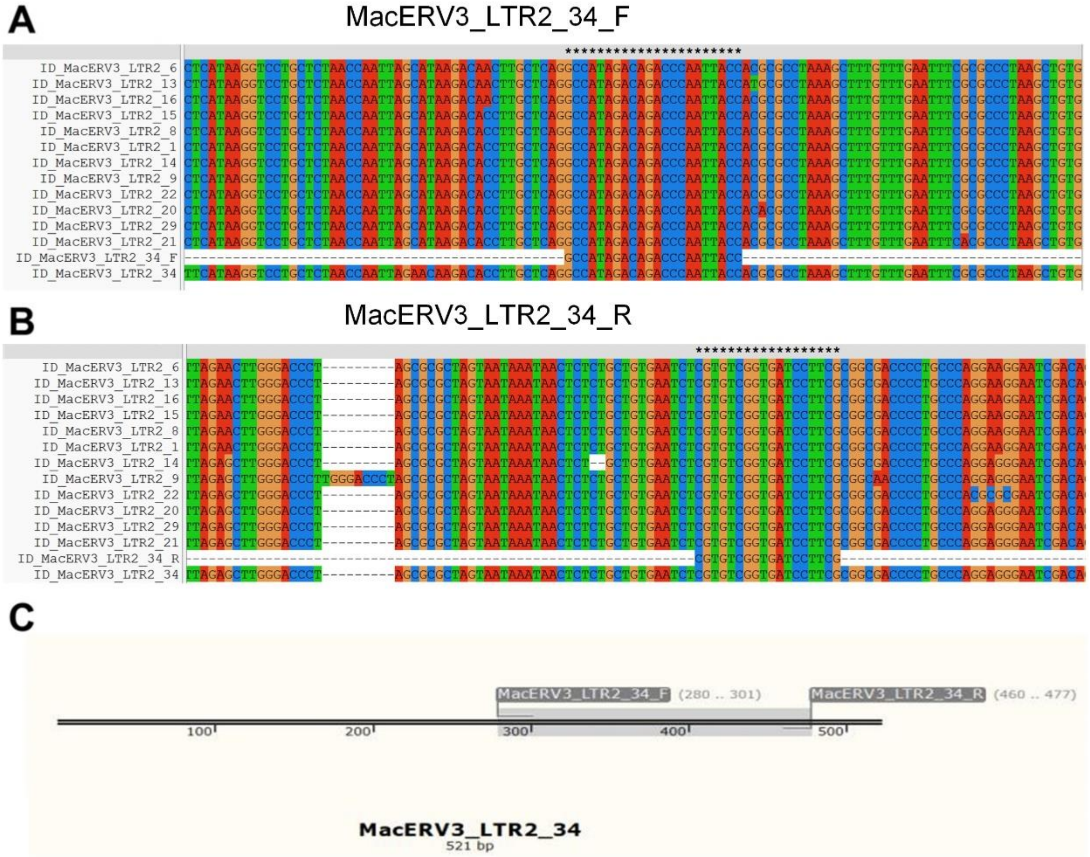

Figure S3. The primers used to validate the expression of 13 MacERV3 LTRs. (A) The alignment of the forward primer MacERV3_LTR2_34_F and the 13 MacERV3 LTRs. (B) The alignment of the reverse primer MacERV3_LTR2_34_R and the 13 MacERV3 LTRs. (C) The precise loci of the two primers on one of the selected MacERV3 LTRs, MacERV3_LTR2_34.
